# Ultra-Orthodox Women in the Job Market: What Helps Them to Become Healthy and Satisfied?

**DOI:** 10.3390/ijerph19138092

**Published:** 2022-07-01

**Authors:** Tehila Kalagy, Sarah Abu-Kaf, Orna Braun-Lewensohn

**Affiliations:** 1 Department of Public Policy and Management, Ben-Gurion University of the Negev, P.O. Box 635, Beer Sheva 8410501, Israel; 2 Department of Interdisciplinary Studies, Conflict Management & Resolution Program, Ben-Gurion University of the Negev, P.O. Box 635, Beer Sheva 8410501, Israel; aks@bgu.ac.il (S.A.-K.); ornabl@bgu.ac.il (O.B.-L.)

**Keywords:** family quality of life, community sense of coherence, diversity climate, inclusive leadership, job satisfaction, mental health

## Abstract

This study explored the mental health and job satisfaction of Ultra-Orthodox women who work in different cultural environments. Data were gathered from 304 Ultra-Orthodox women who belong to various streams in this society and who were recruited by the Midgam research panel. The participants filled out self-reported questionnaires that assessed their family quality of life, community sense of coherence, diversity climate, inclusive leadership, job satisfaction, and mental health. The participants ranged in age between 19 and 64 years (*M* = 30.86, *SD* = 8.71); 43.1% worked within the Ultra-Orthodox enclave, while 22.4% worked with both Ultra-Orthodox and secular individuals and 34.5% worked in mainly secular environments. We observed differences and similarities among the three groups of women. Community sense of coherence was weakest among those who worked outside the enclave, while diversity perception and inclusive leadership were highest among that group. In all three groups, family and community were the most important resources for mental health. Both traditional resources (i.e., family and community) and other resources (i.e., perception of diversity climate and inclusive leadership) were important for job satisfaction.

## 1. Introduction

Culture and ethnicity are crucial to our identity and our perceptions of ourselves and others. They are also responsible for our perception of health, values, and vitality [1]. Thus, it seems important to examine the consequences of human diversity among several cultural and ethnic groups who live in the same country (i.e., Ultra-Orthodox) [2,3] and share a work sphere/environment. More specifically, in the present study, we explored Ultra-Orthodox women’s mental health and the satisfaction they derive from their work. The focus on these women may teach us about women from other minority groups around the world, who are integrating into the general labor market. It is important to study the integration of these women into the workforce, as they are members of minority groups who experience significant difficulties in that integration, due to cultural differences and feelings of foreign-ness [2,3].

Drawing on the ecological model [4], we wanted to understand the ways in which the different circles in a woman’s life impact her mental health and satisfaction from work. Specifically, we were interested in examining family, communal, and work variables as potential explanatory variables of mental health and job satisfaction among Ultra-Orthodox women who work in three different types of environments: within Ultra-Orthodox society (within the enclave), outside the Ultra-Orthodox enclave (i.e., working mostly with people who are not Ultra-Orthodox), and in mixed work environments that include individuals who belong to Ultra-Orthodox society, as well as individuals who belong to secular or national-religious communities.

### 1.1. Literature Review

#### 1.1.1. The Ecological Model

**Figure 1:**
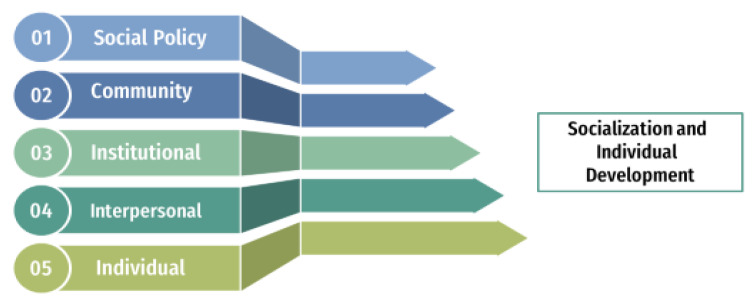
The ecological model.

The present research is based on the ecological model (See Figure 1) [4], which claims that socialization and individual development are influenced by several circles with which one is in communication. It is a theoretical framework that considers the resources of the individual that are present in his/her surroundings, that is, his/her family, his/her community, work environment, etc. In each circle, the individual is an active actor who is influenced by others and who also influences others in that circle. Thus, s/he learns to adapt to the circle’s rules, conditions and restrictions. The different circles—the microsystem, mesosystem, exosystem, and macrosystem—interact with each other. Therefore, the ecological model can serve as an important tool when considering the optimal integration of women from cultural minorities into the workforce, as these circles may help individuals to cope with stress and conflicts in the workplace.

#### 1.1.2. Microsystem—Family Quality of Life

Family and work conflicts arise when there is no balance between an individual’s roles at work and his/her roles outside of work (i.e., in the family) [5]. Although many studies have examined the effects of family–work conflicts on job satisfaction, several studies have focused on a different angle, namely, how family resources help individuals to perform better at work, thereby enhancing the satisfaction that they acquire from their work, as well as their mental health [6,7]. The present study will employ the resources approach to examine how the core resource of family quality of life (i.e., family’s strength, family’s communication, etc.) is related to community and work resources, with the aim of assessing its potential ability to enhance individuals’ job satisfaction and mental health.

#### 1.1.3. Mesosystem—Community Sense of Coherence

Community sense of coherence includes the perception of the community with regard to the three components of Antonovsky’s concept of sense of coherence [8]: comprehensibility (i.e., resources in the community that advance the sense that life in the community is predictable and safe), manageability (i.e., resources that can assist individuals in times of crisis and distress), and meaningfulness (i.e., resources that enable individuals to express and to actualize themselves and to feel satisfaction, challenge, and interest) [9]. The assumption is that, in all cultures, individuals who view their community as being comprehensible, manageable, and meaningful will have another resource to rely on when needed. In the context of the present research, it is important to note that previous studies have found that higher levels of community sense of coherence can contribute to greater job satisfaction and are also associated with better subjective well-being (e.g., [10,11]). Specifically in the cultural group that is the subject of the present study (i.e., the Ultra-Orthodox), community sense of coherence has been shown to be an important contributor to job satisfaction and well-being [12]. The present study will examine the role of community sense of coherence as it relates to additional factors in the job environment, namely, diversity perceptions and inclusive leadership, which could all explain the job satisfaction and well-being of Ultra-Orthodox women in their work environments.

#### 1.1.4. Exosystem—Diversity Perceptions and Inclusive Leadership

Diversity perception is a source of learning and creativity that helps to create opportunities for employees’ development and promotion, as well as organizational profitability [13]. Studies have found that workplaces that promote diversity experience better decision-making, provide better service to various ethnic groups, and enjoy productivity [14,15]. Cultural diversity in the workplace is of great interest for research and public administration. Despite the above-mentioned benefits, diversity in the workplace appears difficult to apply and is also associated with some negative outcomes, such as resistance from employees to working in teams that include members of various ethnic groups [16,17,18]. However, in general, diversity and diversity management generate positive outcomes for organizations. They produce new ideas and enhance the ability of employees at various stages and statuses to grow, learn, and imagine new possibilities. In addition, diverse workplaces tend to have relatively low levels of discrimination [19]. Diversity adds important value to the workplace when it is managed effectively and yields a higher quality of work because of the broad perspectives and ideas for problem-solving and the solutions that arise [20,21].

Inclusive leadership involves the openness, accessibility, and availability of the leader (i.e., the boss or supervisor) for discussion with his/her employees. The various aspects of inclusive leadership in a work environment include the boss’s care for his/her employees, the communication of desirable expectations, and new opportunities for individual employees, as well as for the organization [22]. The setting of goals by bosses and supervisors together with employees can improve an organization’s accomplishments. This means that the leaders expect and support their workers in initiating efforts to solve various problems that arise and, in that way, give the team members an appropriate voice [23]. This promotes employee trust and loyalty, which is reflected by the recognition, responsiveness, and responsibilities of bosses and supervisors [24,25]. Awareness, listening, and acting fairly toward employees and their needs are additional important components of inclusive leadership. All of these behaviors develop a social context that is safe for employees, which allows employees to speak up and contribute their input in different areas of the work sphere. In this way, the inclusive leader contributes to employees’ job satisfaction and well-being [26].

### 1.2. Outcomes: Job Satisfaction and Mental Health

#### 1.2.1. Job Satisfaction

Job satisfaction has important implications for employee well-being [27] that derive from a need to believe that one’s actions within the framework of one’s work are important and significant [28]. One of the definitions of job satisfaction focuses on the perceptions of fulfilment as a result of day-to-day activities. This fulfilment is associated with job commitment, as well as higher levels of performance at work [29]. It seems that one of the contributors to greater job satisfaction is high income [30]. In the present research, we sought to examine women from a unique minority group, (i.e., the Ultra-Orthodox) and the way in which the above-mentioned variables, from different ecological circles, contribute to their job satisfaction. Community sense of coherence attributed to the community from which the minority individual comes has been reported only in one study, in which it was found to be a limited explanatory factor of job satisfaction [12]. Diversity perception [31] and inclusive management [26] have been reported to have positive effects on job satisfaction.

#### 1.2.2. Mental Health

Positive mental health or well-being is important in the work sphere. Those who report positive mental health have better work performance and also enjoy better social relations and physical health [32]. Positive work environments also contribute to employee mental health, which, in turn, benefits their organizations [33]. Thus, it seems that there are strong positive relationships between job satisfaction and the physical and mental health of employees [34]. Workplace practices are important contributors to a mentally healthy worker [35].

### 1.3. Research Questions

In line with the literature reviewed above and our research aims, several research questions were formulated:

1. Are there differences between Ultra-Orthodox women who work within the Ultra-Orthodox enclave, those who work with the Ultra-Orthodox sector and other sectors of Israel society, and those who work mainly outside the Ultra-Orthodox enclave, in terms of family quality of life, community sense of coherence, diversity perception, inclusive leadership, job satisfaction, and/or mental health [2,3]?
2. Based on the ecological model that evaluates the different circles of one’s life as promoting one’s mental health, we built a model that included the main study variables: Family Quality of Life, Community Sense of Coherence, Diversity Perceptions subscales, and Inclusive Leadership subscales as potential explanatory factors of employees’ satisfaction from work and their mental health. The two dependent variables (i.e., job satisfaction and mental health) were examined separately [4,5,8].

This full model was tested, in our effort to identify the similarities and differences among the three groups of Ultra-Orthodox women: those who work within the ultra-Orthodox enclave, those who work with both the Ultra-Orthodox sector and other sectors, and those who work mainly with other sectors.

## 2. Materials and Methods

### 2.1. Participants

Three hundred and four Ultra-Orthodox women aged 19–64 (*M* = 30.86, *SD* = 8.71) participated in this study. Among the participants, 195 (64.1%) had earned an academic degree and 109 (35.9%) had completed studies for a certificate beyond a high school diploma. One hundred and eighty-five participants (60.9%) reported that they worked fully in the profession for which they had studied, 68 (22.4%) partially worked in their professions, and 51 (16.8%) did not work in the profession they had studied. One hundred and thirty-one participants (43.1%) worked within the Ultra-Orthodox enclave, 68 (22.4%) reported that they worked in a mixed environment, and 105 (34.5%) worked in a mostly secular environment. With regard to socioeconomic status, 213 (70%) reported a lower than average income, 43 (14.1%) reported an average income, and 15 (4.9%) reported a higher than average income. In addition, 30 participants preferred not to reveal their income and 3 (who were not included in further analyses) reported that they did not have any income at all. Most of the participants were married (85.9%) and reported having 0–11 children (*M* = 2.78, *SD* = 2.37). They belonged to various streams of Israeli Ultra-Orthodox society: 50.7% identified with the Lithuanian stream, 21.4% as Hasidic, 22.7% as Mizrachi, 4.3% as Modern, and 1% as others.

### 2.2. Procedure

After receiving ethical approval (no. 1739-2), data were gathered via the Midgam platform (https://www.midgampanel.com/; accessed on 24 December 2020) an internet platform for the distribution of questionnaires). Participants received a written explanation about the study and its aim and were asked to sign an informed-consent form. After signing that form, they were transferred to the questionnaire site, which kept their answers anonymous. Participants were free to withdraw their participation for any reason and at any time during the questionnaire procedure.

### 2.3. Measures

#### 2.3.1. Demographic Data

Demographic background data included gender, age, level of education, socioeconomic status, and whether or not participants were working in the profession for which they had trained. Additionally, participants reported whether they worked in a mostly secular environment, in a mixed environment, or within the Ultra-Orthodox enclave.

#### 2.3.2. The Family Quality of Life Scale (FQOL)

This construct includes 25 items, which are rated using a 5-point Likert-type scale ranging from 1 (*not at all satisfied*) to 5 (*very satisfied*). The questionnaire focuses on the satisfaction of the different members of the family with the family’s quality of life [36].

The dimensions include interactions between family members, parenthood, emotional wellness, physical and material wellness, etc. The higher the score, the better the quality of family life. Sample item: *To what extent are you satisfied with how open family members are with one another?* The Cronbach alpha reliability coefficient for the current sample was 0.94.

#### 2.3.3. Sense of Community Coherence 

This construct includes 12 items, which are each rated on a 7-point Likert-type scale with anchoring phrases at each end. It translates the major themes of Antonovsky’s [8] personal sense of coherence—comprehensibility, manageability, and meaningfulness—into community resources. Examples of the items: *To what extent do you feel that you have influence in your community?; I intend to live in this community in the future* [37].

The Cronbach alpha coefficient for the present study was 0.88.

#### 2.3.4. Diversity-Perceptions Scale 

This construct includes 16 items, which are rated on a 6-point Likert-type scale with anchoring phrases at each end (1—*do not agree at all*; 6—*definitely agree*.) The higher the score, the more openness to diversity there is at the organization [38]. In addition to the full scale, we also calculated mean scores for three subscales after reversing the relevant items. Those three subscales were: Organization’s Fairness [example: *I feel that I have been treated differently here because of my race, gender, religion or age* (reversed)], Organizational Inclusion [example: *Management here encourages the formation of employee network support groups*], and Personal Diversity Values [example: *Knowing more about cultural norms of diverse groups would help me to be more effective in my job*]. The Cronbach alpha reliability coefficients for the full scale and the subscales ranged between 0.73 and 0.82.

#### 2.3.5. Inclusive Leadership Scale (ILS) 

This construct was used to evaluate the relationships between managers and their workers. The evaluation included dimensions such as respect, awareness, and recognition of the worker and his/her contribution to the organization, fairness, etc. [22,39]. The questionnaire includes 16 items, which are each rated on a 5-point Likert-type scale. In addition to the full scale, we also used three subscales in this study: Support Recognition (example: *Asks for my ideas about my work*), Communication-Action-Fairness (example: *Concerned with how things are, or are not, being done*); Self-Interest–Disrespect (example: *Makes comments to put me down*). The items for the Self-Interest–Disrespect subscale were reversed, so that they would represent the respect angle of inclusive leadership. Means were calculated for each scale and alpha reliability coefficients ranged between 0.76 and 0.89.

#### 2.3.6. Employee Satisfaction Inventory (ESI) 

The employee satisfaction inventory is a 24-item inventory in which the items are each rated on 5-point Likert scale ranging from 1 (*strongly agree*) to 5 (*strongly disagree*). The inventory includes six subscales: working conditions, supervisors, pay, the job itself, the organization as a whole, and promotion. The global ESI scale is derived from the mean score of all items [27]. 

The psychometrics of the scale and the subscales have been shown to be reliable [27]. In the present study, the Cronbach alpha coefficient for the global scale was 0.90.

#### 2.3.7. General Health Construct (GHQ-12)

This questionnaire was used to evaluate mental health [40,41].

It includes 12 items that are each rated on a 5-point Likert-type scale ranging from 1 (*very often*) to 5 (*never*). The higher the score, the better one’s mental health. The questionnaire includes items such as: *To what extent do you feel that you can handle your problems?* The mean of the scores for the 12 items was calculated and the Cronbach’s alpha reliability coefficient was 0.89.

### 2.4. Data Analyses

First, one-way ANOVA was performed to explore differences between the three groups of workers in terms of all of the study variables. Then, an AMOS model was used to evaluate the explanations of the two dependent variables (i.e., job satisfaction and mental health) by the main study variables from the different ecological systems. Finally, we used a nested model to evaluate the different effects of the main study variables on both job satisfaction and mental health, to explore different effects among the different groups of workers.

## 3. Results

The first question, which examined differences between the three groups of workers, was explored using one-way ANOVA. The results of that analysis are presented in Table 1.

As shown in Table 1, several differences emerged between the three groups of women. The women who worked in mixed environments reported less of a diversity climate, in terms of diversity perception and fairness, and less inclusive leadership, support recognition, and respect from their bosses/supervisors. Those who worked outside the enclave reported the weakest community sense of coherence when relating to the Ultra-Orthodox community. The strongest community sense of coherence was reported by those women who worked within the enclave.

We used a multi-group analysis to compare the effects of the different ecological factors on each group of women (i.e., those working within the enclave, those working with the Ultra-Orthodox sector and other sectors, and those working mainly with other sectors), in terms of the two dependent variables. The means for each scale were computed separately and used as manifest variables. The two dependent variables, job satisfaction and mental health, were presented as manifest variables. In the final model, job satisfaction played a role as an explanatory variable for the second dependent variable (mental health), in addition to the other independent variables in the ecological model. Model fit was assessed using the ratio of chi-square to degrees of freedom (χ^2^/*df*) incremental fit index (IFI [42], the comparative fit index (CFI [43]), and the root mean square error of approximation (RMSEA [44]).

**Figure 2:**
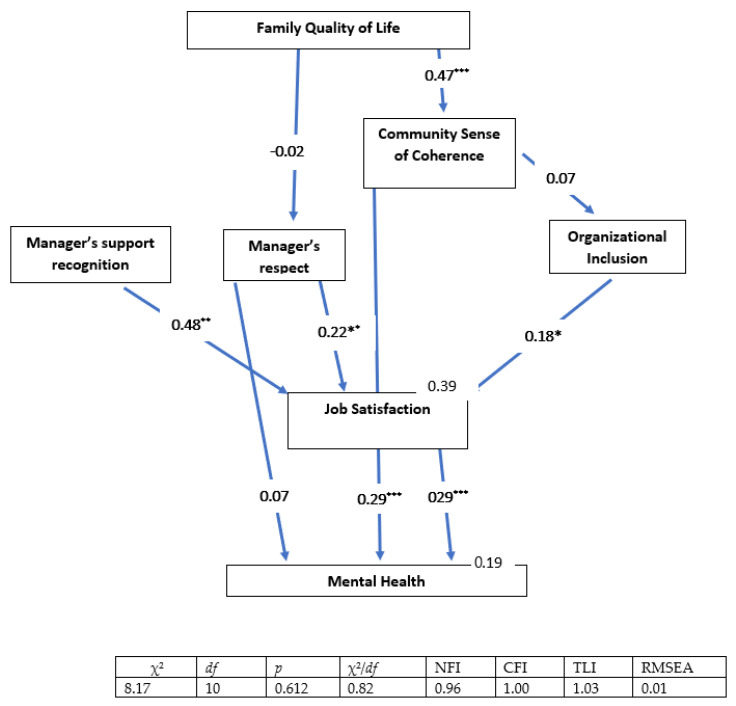
The total direct and indirect effects of the different factors (family quality of life, community sense of coherence, manager’s support recognition, manager’s respect, and organizational inclusion) on job satisfaction and mental health among the women who worked within the enclave. All of the coefficients in the figure are standardized. Goodness-of-fit indices are presented below. * *p* < 0.05; ** *p* < 0.01; *** *p* < 0.001.

**Figure 3:**
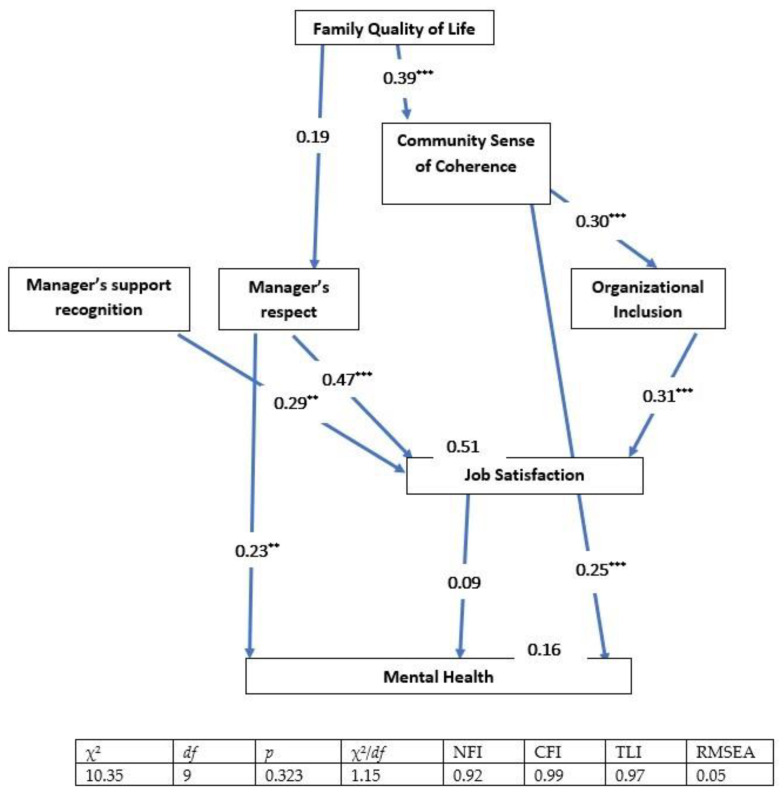
The total direct and indirect effects of the different factors (family quality of life, community sense of coherence, manager’s support recognition, manager’s respect, and organizational inclusion) on job satisfaction and mental health among the women who worked in a mixed environment. All of the coefficients in the figure are standardized. Goodness-of-fit indices are presented below. ** *p* < 0.01; *** *p* < 0.001.

**Figure 4:**
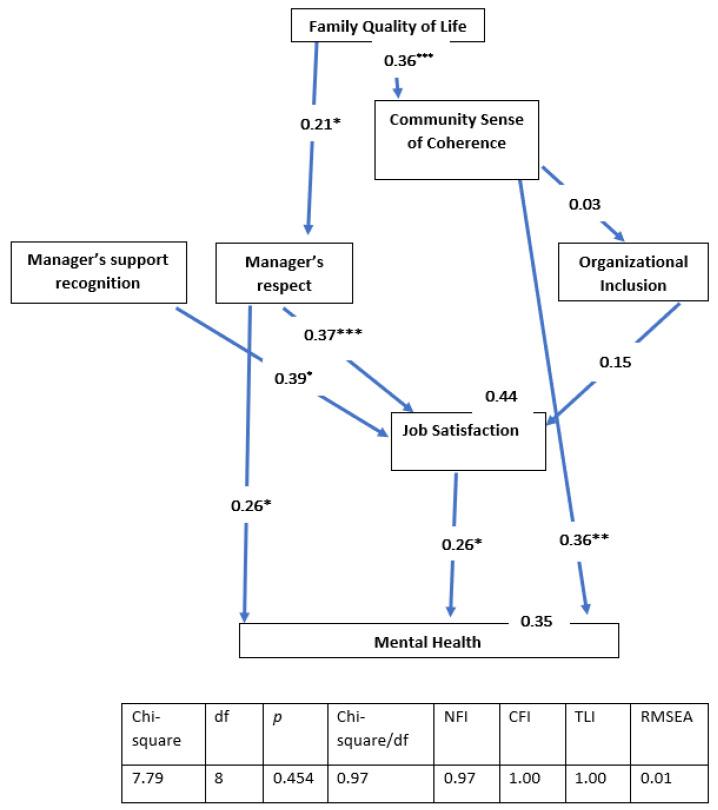
The total direct and indirect effects of the different factors (family quality of life, community sense of coherence, manager’s support recognition, manager’s respect, and organizational inclusion) on job satisfaction and mental health among the women who worked outside the enclave. All of the coefficients in the figure are standardized. * *p* < 0.05; ** *p* < 0.01; *** *p* < 0.001.

Acceptable fit was indicated by a χ^2^/*df* ratio of 3 or less [45], IFI and CFI values equal to or greater than 0.90, and an RMSEA value of less than 0.08 [44,46]. The indices for the overall model were as follows: χ^2^_(27)_ = 93, *p* < 0.001; χ^2^/*df* = 3.45; CFI = 0.85; IFI = 0.86; and RMSEA = 0.09 (Figure 2, Figure 3 and Figure 4). The inadequate parameters could be due to significant differences between the three groups. The parameters were computed separately for each group and, in each case, they were found to be adequate. These parameters are presented below as part of the figures describing each group.

The final SEM presents only relationships that were significant among at least one of the groups; variables that were not significant for the explanation of job satisfaction and/or mental health were deleted, in order to streamline the presentation. The overall model explained 39% of the variance in job satisfaction among the women who worked within the enclave, 51% of the variance among the women who worked in a mixed environment, and 44% of the variance among the women who worked outside the enclave. As for mental health, the overall model explained 19% of the variance in mental health among the women who worked within the enclave, 16% of the variance among the women who worked in a mixed environment, and 35% of the variance among the women who worked mainly with other sectors (i.e., outside the enclave).

The indirect effects of the various variables on job satisfaction were as follows. Among the women who worked within the enclave: Family Quality of Life (0.02) and Community Sense of Coherence (0.01). Among the women who worked in mixed environments: Family Quality of Life (0.12) and Community Sense of Coherence (0.09). Finally, among the women who worked mainly with other sectors: Family Quality of Life (0.08) and Community Sense of Coherence (0.01).

The indirect effects of the various variables on mental health were as follows. Among the women who worked within the enclave: Family Quality of Life (0.14), Respect (0.06), Organizational Inclusion (0.05), and Support Recognition (0.14). Among the women who worked in mixed environments: Family Quality of Life (0.15), Community Sense of Coherence (0.08), Respect (0.04), Organizational Inclusion (0.03), and Support Recognition (0.03). Finally, among women who worked mainly with other sectors: Family Quality of Life (0.20), Respect (0.09), Organizational Inclusion (0.04), and Support Recognition (0.10).

The total direct and indirect effects of the different factors on job satisfaction varied among the three groups of women. Among the women who worked within the enclave, Support Recognition had the most powerful effect (0.48), followed by Respect (0.22), Organizational Inclusion (0.18), and Community Sense of Coherence (0.14). Among the women who worked in mixed environments, Respect had the most powerful effect (0.47), followed by Organizational Inclusion (0.31) Support Recognition (0.29), Family Quality of Life (0.13) and Community Sense of Coherence (0.09). Finally, among the women who worked mainly outside the enclave, Support Recognition had the most powerful effect (0.39), followed by Respect (0.37), Organizational Inclusion (0.15) and Family Quality of Life (0.08).

The direct and indirect effects of the different factors on mental health also varied between the three groups of women. Among the women who worked within the enclave, Job Satisfaction, Community Sense of Coherence, and Family Quality of Life had equally powerful effects on mental health (all 0.29), with weaker effects observed for Support Recognition (0.14), Respect (0.14), and Organizational Inclusion (0.05). Among the women who worked in a mixed environment, Respect had the most powerful effect on mental health (0.27), followed by Community Sense of Coherence (0.26) and Family Quality of Life (0.15); Support Recognition and Respect had effects that were similar to one another in strength (0.03). Finally, among those who worked mainly outside the enclave, there were two pairs of variables with similar indirect effects on mental health: Community Sense of Coherence and Respect (both 0.36) and Job Satisfaction and Respect (both 0.16).

These results indicate that there were major differences between the three groups. To test whether these major differences in the strength of the relationships between the various independent and the dependent variables were significant, the effects of the independent variables on job satisfaction and mental health were examined using a nested model. Equality constraints among groups were assigned for each effect, to allow for the comparison of the constrained model with the free model. Statistical differences for Job Satisfaction as a dependent variable were found for the variables as follows: Organizational Inclusion [(χ^2^_(27)_ = 93.2); Δχ^2^_(3)_ = 412.4; *p* ≤ 0.001], Support Recognition [(χ^2^_(27)_ = 93.2); Δχ^2^_(3)_ = 284.4; *p* ≤ 0.001], and Respect [(χ^2^_(27)_ = 93.2); Δχ^2^_(3)_ = 272.5; *p* ≤ 0.001].

Statistical differences for the model in which mental health was the dependent variable were found for the variables as follows: Community Sense of Coherence [(χ^2^_(27)_ = 93.2); Δχ^2^_(3)_ = 558.3; *p* ≤ 0.001], Respect [(χ^2^_(27)_ = 93.2); Δχ^2^_(3)_ = 435.6; *p* ≤ 0.001], and Job Satisfaction [(χ^2^_(27)_ = 93.2); Δχ^2^_(3)_ = 383.5; *p* ≤ 0.001]. This means that despite the fact that most of the examined variables made important and significant contributions to job satisfaction and mental health among the different groups, they differed significantly in the strength of their contributions to those outcomes among the different groups.

Power analysis for the proposed model was conducted using the MacCallum et al. [47] approach, with effect size defined as the difference in model Root Mean Square Error of Approximation (RMSEA) between close fit (RMSEA < 0.05) and the lack of fit (RMSEA > 0.10). Given α = 0.05 and a sample size of *n* = 304, power was 0.98, which is above the recommended cutoff of 0.80.

## 4. Discussion

The aim of the present study was to explore the mental health of Ultra-Orthodox women and the satisfaction that they derive from their work. We wanted to understand how family, communal, and work resources explain mental health and job satisfaction. Drawing on the ecological model, we chose variables from various circles of one’s life. First, we compared family quality of life, community sense of coherence, diversity perception, and inclusive leadership, as well as job satisfaction and mental health across three groups of Ultra-Orthodox women (i.e., those working within the enclave, those working in mixed environments, and those working outside the enclave). While we found no differences in family quality of life, job satisfaction. or mental health across the three groups, we did find differences in community sense of coherence, diversity perception, and inclusive leadership. The weakest community sense of coherence was reported by those who worked outside the enclave [12]. The results presented here expand upon that finding and show that women who work outside the enclave experience similar feelings and perceptions and, such as those men, do not feel that they can influence their community as much as those who work and stay only within the enclave.

The other two variables, diversity climate perception and inclusive leadership, were weakest among the Ultra-Orthodox women who work with both the Ultra-Orthodox sector and other sectors of Israeli society. It seems that working with individuals from different sectors can be confusing and that confusion can be reflected in constant comparisons with the ‘other’, which might result in a feeling that there is not enough room for and openness to the special population of Ultra-Orthodox women. This, in turn, is reflected by the two variables of diversity perception and inclusive leadership.

Our main question was related to how family quality of life, community sense of coherence, diversity perception, and inclusive leadership in the workplace explain mental health and job satisfaction. To that end, we built a model to evaluate the direct and indirect effects of these variables on job satisfaction and mental health. We found similarities, as well as differences in the ways that the different variables explained the dependent variables among the three groups of women. When we looked at mental health as the dependent variable, we found that community sense of coherence was important explanatory factors of mental health among all three groups of women. It seems that in a traditional society such as Ultra-Orthodox society, women need the support of their community and recognition to go out to work in any type of work environment. Moreover, the belief that their community have the necessary resilience and resources to cope with the challenges contributes significantly to these women’s mental health.

The main difference that emerged in the explanation of mental health was in job satisfaction, which was an important contributor to mental health for those who worked within and outside the enclave, but was not significant among the women who worked in a mixed environment. While the findings regarding the groups of women who worked within and outside the enclave resembled those of other studies on different populations around the world in which job satisfaction has been shown to have a positive impact on the physical and mental health of employees [34], it seems that, in the mixed group, only specific workplace practices, such as respect from managers/supervisors, make important contributions to the mental health of Ultra-Orthodox women workers [35].

The picture for job satisfaction is a bit more complicated. When we look at the three main factors that explain job satisfaction, we can see that manager’s support/recognition and respect, which are two main aspects of inclusive leadership, were important for all three groups of employees. Indeed, support/recognition and respect, which includes supervisors asking their employees for their ideas, recognizing their contributions to the organization, encouraging them to be involved, letting them make decisions about things related to their jobs, showing interest in what their employees are doing, giving them credit for what they are doing, and making positive comments, have been found to be important factors for job satisfaction in various populations [26]. It seems that inclusive leadership and its contribution to job satisfaction remain important among Ultra-Orthodox women. These findings point to the importance of those circles in a traditional closed society, that grant legitimacy and power to those who choose to go out of the enclave to experience a more challenging work environment.

### 4.1. Study Limitations

Information about personal work experiences was provided only by the individuals themselves and, therefore, the collected data are subjective. Despite this limitation, the importance of this study lies in the fact that it is a field study, with the field providing a natural laboratory for the investigation of human behavior [48]). An additional limitation is the size of the mixed-environment group, which was smaller than the other two groups. Therefore, we believe that future studies should focus on this group, to provide a more thorough understanding of the experiences of those who work in such environments.

### 4.2. Study Strengths

The importance of this study lies in the fact that it is a field study rooted in a theoretical model. This study is focused on an important minority (women) within a minority population (Ultra-Orthodox) that is growing and now accounts for about 12.6% of the entire Israeli population. We examined Ultra-Orthodox women in the work sphere, distinguishing between three unique groups that are rarely studied: those who work within the Ultra-Orthodox enclave, those who work in a mixed environment, and those who work outside the enclave. The results of this study have important implications for public policy.

## 5. Conclusions

This study aimed to investigate the experiences of Ultra-Orthodox women in the work environment. To that end, we studied three groups of Ultra-Orthodox women: those who work within the Ultra-Orthodox enclave, those who work outside the Ultra-Orthodox enclave, and those whose work sphere is mixed. First, we compared the three groups in terms of the different study variables and found differences in community sense of coherence, diversity perception, and inclusive leadership, with those working outside the enclave reporting the weakest community coherence and the highest perceived levels of diversity climate and inclusive leadership in their work environments.

Our model that included both job satisfaction and mental health revealed significant similarities between these groups of women. Family and community are important resources for the mental health of women in all three groups. We also found that both the traditional resources of family and community and resources of the job environment (i.e., perception of diversity climate and inclusive leadership) are all important, to varying degrees, as explanatory factors of job satisfaction.

In conclusion, we make two key policy recommendations for the integration of women from minority groups into the general workforce. First, in light of the finding that community sense of coherence and family quality of life are important explanatory factors for the mental health of all three groups of women, we recommend focusing on those two factors. The intention is to establish legitimacy among the family and the community regarding Ultra-Orthodox women going out to work, through the enlistment of rabbinical leadership and influencers in the community. Expanding legitimacy for this professional activity and establishing a relationship with the community would significantly assist Ultra-Orthodox women in their integration into the workforce and would positively contribute to their mental health.

We also recommend the development of unique training programs for employers, to provide them with tools for integrating minorities into their organizations. As we have seen, the main factor contributing to job satisfaction across all three groups was the recognition and respect of one’s manager. This suggests that developing and integrating the concept of inclusive leadership into organizations would lead to better placement of employees belonging to minority groups, increase their job satisfaction, and, ultimately, benefit the organization, those integrating into the organization, and society as a whole.

## Figures and Tables

**Table 1:** Differences between Ultra-Orthodox women who (a) work only within the enclave, (b) work with both the Ultra-Orthodox sector and other sectors, and (c) work outside the enclave.

Variables	Within the Enclave ^(a)^ *N* ≈ 131		Both ^(b)^ *N* ≈ 68		Outside the Enclave ^(c)^ *N* ≈ 105		*F*
	*M*	*SD*	*M*	*SD*	*M*	*SD*	
Family quality of life (1–5)	4.00	0.61	4.02	0.64	3.87	0.65	1.76
Community sense of coherence (1–7)	5.30	0.92	5.08	1.05	4.98	1.10	3.10 *^(ac)^
Diversity perceptions (1–6)	4.26	0.73	4.06	0.75	4.33	0.69	2.96 ^^(bc)^
Organization fairness (1–6)	4.75	1.14	4.34	1.31	4.70	1.12	2.91 ^^(ab)^
Organizational inclusion (1–6)	2.79	1.00	2.67	0.93	2.90	0.90	1.16
Personal diversity values (1–6)	4.01	1.25	4.03	1.45	4.31	1.24	1.69
Inclusive leadership (1–5)	3.77	0.65	3.61	0.72	3.90	0.65	4.08 *^(bc)^
Support-Recognition (1–5)	3.70	0.95	3.55	0.86	3.89	0.85	3.17 *^(bc)^
Communication-Action-Fairness (1–5)	3.57	0.78	3.55	0.79	3.72	0.71	1.46
Respect for the worker (1–5)	4.06	0.74	3.73	0.91	4.09	0.82	4.05 *^(ab, bc)^
General health (1–5)	3.33	0.48	3.25	0.55	3.20	0.52	1.95
Work satisfaction (1–5)	3.58	0.73	3.42	0.77	3.63	0.79	1.62

* *p* < 0.05; ^ *p* = 0.05.

## Data Availability

Data collected and used in this study are available from the authors upon reasonable request.

## References

[1] Sue D.W., Sue D. (2013). Counseling the Culturally Diverse: Theory & Practice.

[2] Chang E.C., Banks K.H. (2007). The color and texture of hope: Some preliminary findings and implications for hope theory and counseling among diverse racial/ethnic groups. Cult. Divers. Ethn. Minority Psychol..

[3] Youssef-Morgan C.M., Hardy J. (2014). A positive approach to multiculturalism and diversity management in the workplace. Perspectives on the Intersection of Multiculturalism and Positive Psychology.

[4] Bronfenbrenner U. (1979). The Ecology of Human Development: Experiments by Nature and Design.

[5] Greenhaus J.H., Beutell N.J. (1985). Sources of conflict between work and family roles. Academ. Manag. Rev..

[6] Carlson D.S., Hunter E.M., Ferguson M., Whitten D. (2014). Work–family enrichment and satisfaction: Mediating processes and relative impact of originating and receiving domains. J. Manag..

[7] Chan X.W., Kalliath T., Brough P., Siu O.L., O’Driscoll M.P., Timms C. (2016). Work–family enrichment and satisfaction: The mediating role of self-efficacy and work–life balance. Int. J. Human Resour. Manag..

[8] Antonovsky A. (1987). Unraveling the Mystery of Health: How People Manage Stress and Stay Well.

[9] Elfassi Y., Braun-Lewensohn O., Krumer-Nevo M., Sagy S. (2016). Community sense of coherence among adolescents as related to their involvement in risk behaviors. J. Community Psychol..

[10] Braun-Lewensohn O. (2015). Inclusion in Israel: Coping resources and job satisfaction as explanatory factors of stress in two cultural groups. J. Res. Spec. Educ. Need..

[11] Braun-Lewensohn O., Bar R. (2017). Coping and quality of life among military wives following a military operation. Psychiatry Res..

[12] Braun-Lewensohn O., Kalagy T. (2019). Between the inside and the outside world: Coping of Ultra-Orthodox individuals with their work environment after academic studies. Community Ment. Health.

[13] De Meuse K.P., Hostager T.J. (2001). Developing an instrument for measuring attitude for and perceptions of workplace diversity: An initial report. Human Resour. Dev. Q..

[14] Cox T. (1991). The multicultural organization. Acad. Manag. Perspect..

[15] Van Knippenberg D., De Dreu C.K., Homan A.C. (2004). Work group diversity and group performance: An integrative model and research agenda. J. Appl. Psychol..

[16] Antwi-Boasiako K.B. (2008). The dilemma of hiring minorities and conservative resistance: The diversity game. J. Instruct. Psychol..

[17] Thomas K.M., Plaut V.C., Thomas K.M. (2008). The many faces of diversity resistance in the workplace. Diversity Resistance in Organizations.

[18] Van Knippenberg D., Schippers M.C. (2007). Work group diversity. Annu. Rev. Psychol..

[19] Friedman H.H., Friedman L.W., Amoo T. (2002). Using humor in the introductory statistics course. J. Stat. Educ..

[20] Hofhuis J., van der Zee K.I., Otten S. (2015). Measuring employee perception on the effects of cultural diversity at work: Development of the benefits and threats of diversity scale. Qual. Quant..

[21] Phillips L.T., Slepian M.L., Hughes B.L. (2018). Perceiving groups: The people perception of diversity and hierarchy. J. Pers. Soc. Psychol..

[22] Carmeli A., Palmon R., Ziv E. (2010). Inclusive leadership and employee involvement in creative tasks in the workplace: The mediating role of psychological safety. Creativ. Res. J..

[23] Meyer D. (2006). Setting the Table.

[24] Hollander E.P. (2008). Inclusive Leadership: The Essential Leader-Follower Relationship.

[25] Hollander E.P., Park B.B., Elman B., Ignagni M.E. (2008). Inclusive leadership and leader-follower relations: Concepts, research, and applications. Memb. Connect. Int. Lead. Assoc..

[26] Choi S.B., Tran T.B.H., Kang S.W. (2017). Inclusive leadership and employee well-being: The mediating role of person-job fit. J. Happiness Stud..

[27] Koustelios A.D., Bagiatis K. (1997). The Employee Satisfaction Inventory (ESI): Development of a scale to measure satisfaction of Greek employees. Educ. Psychol. Meas..

[28] Malach Pines A. (2002). A psychoanalytic-existential approach to burnout: Demonstrated in the cases of a nurse, a teacher, and a manager. Psychother. Theory Res. Pract. Train..

[29] Judge T.A., Thoresen C.J., Bono J.E., Patton G.K. (2001). The job satisfaction–job performance relationship: A qualitative and quantitative review. Psychol. Bull..

[30] Dolan P., Peasgood T., White M. (2008). Do we really know what makes us happy? A review of the economic literature on the factors associated with subjective well-being. J. Econ. Psychol..

[31] Madera J.M., Dawson M., Guchait P. (2016). Psychological diversity climate: Justice, racioethnic minority status and job satisfaction. Int. J. Contemp. Hosp. Manag..

[32] Huppert F.A. (2009). Psychological well-being: Evidence regarding its causes and consequences. Appl. Psychol. Health Well-Being.

[33] Dutton J.E., Roberts L.M., Bednar J., Donaldson S.I., Csikszentmihalyi M., Nakamura J. (2011). Prosocial practices, positive identity, and flourishing at work. Applied Positive Psychology: Improving Everyday Life, Health, Schools, Work, and Society.

[34] Scanlan J.N., Still M. (2019). Relationships between burnout, turnover intention, job satisfaction, job demands and job resources for mental health personnel in an Australian mental health service. BMC Health Serv. Res..

[35] Harvey S., Joyce S., Tan L., Johnson A., Nguyen H., Modini M., Groth M. (2014). Developing a Mentally Healthy Workplace: A Review of the Literature.

[36] Hu X., Summers J.A., Turnbull A., Zuna N. (2011). The quantitative measurement of family quality of life: A review of available instruments. J. Intellect. Disabil. Res..

[37] Braun-Lewensohn O., Sagy S. (2011). Salutogenesis and culture: Personal and community sense of coherence in different cultural groups. Int. Rev. Psychiatry.

[38] Barak M.E., Cherin D.A., Berkman S. (1998). Organizational and personal dimensions in diversity climate: Ethnic and gender differences in employee perceptions. J. Appl. Behav. Sci..

[39] Hollander E.P., Couto R.A. (2007). Relating leadership to active followership. Reflections on Leadership.

[40] Derogatis L.R. (1993). BSI—Brief Symptom Inventory. Administration, Scoring, and Procedures Manual.

[41] Derogatis L.R., Savitz K.L. (2000). The SCL–90–R and Brief Symptom Inventory (BSI) in Primary Care.

[42] Bollen K. (1989). Structural Equations with Latent Variables.

[43] Bentler P. (1990). Comparative fit indexes in structural models. Psychol. Bull..

[44] Browne M.W., Cudeck R., Bollen K.A., Long J.S. (1993). Alternative ways of assessing model fit. Testing Structural Equation Models.

[45] Carmines E.G., McIver J.P., Bohrnstedt G.W., Borgatta E.F. (1981). Analyzing models with unobserved variables. Social Measurement: Current Issues.

[46] Hoyle R.H. (1995). Structural Equation Modeling: Concepts, Issues, and Applications.

[47] MacCallum R.C., Browne M.W., Sugawara H.M. (1996). Power analysis and determination of sample size for covariance structure modeling. Psychol. Methods.

[48] Lazarus R.S. (1982). Thoughts on the relations between emotion and cognition. Amer. Psychol..

